# Zn(II)-Based Mixed-Ligand-Bearing Coordination Polymers as Multi-Responsive Fluorescent Sensors for Detecting Dichromate, Iodide, Nitenpyram, and Imidacloprid

**DOI:** 10.3390/polym15112570

**Published:** 2023-06-02

**Authors:** Dan Wang, Lin-Huan Du, Long Li, Yu-Meng Wei, Tao Wang, Jun Cheng, Bin Du, Yi Jia, Bao-Yi Yu

**Affiliations:** 1 Key Laboratory of Urban Agriculture in North China, Ministry of Agriculture and Rural Affairs, Beijing University of Agriculture, Beijing 102206, China; 202130123127@bua.edu.cn (D.W.); 202120151031@bua.edu.cn (L.-H.D.); 202020152122@bua.edu.cn (Y.-M.W.); 202220151161@bua.edu.cn (T.W.); chengjun@bua.edu.cn (J.C.); 2 Beijing Key Laboratory of Agricultural Product Detection and Control of Spoilage Organisms and Pesticide Residue, Faculty of Food Science and Engineering, Beijing University of Agriculture, Beijing 102206, China; bindu80@bua.edu.cn; 3 Beijing National Laboratory for Molecular Sciences, CAS Key Lab of Colloid, Interface and Chemical Thermodynamics, Institute of Chemistry, Chinese Academy of Sciences, Beijing 100190, China; jiayi@iccas.ac.cn

**Keywords:** coordination polymers, fluorescence sensing, *d*^10^ metal, CP, sensing ions, sensing pesticides

## Abstract

Coordination polymers (CPs) are organo-inorganic porous materials consisting of metal ions or clusters and organic linkers. These compounds have attracted attention for use in the fluorescence detection of pollutants. Here, two Zn-based mixed-ligand-bearing CPs, [Zn_2_(DIN)_2_(HBTC^2−^)_2_] (CP-1) and [Zn(DIN)(HBTC^2−^)]·ACN·H_2_O (CP-2) (DIN = 1,4-di(imidazole-1-yl)naphthalene, H_3_BTC = 1,3,5-benzenetricarboxylic acid, and ACN = acetonitrile), were synthesized under solvothermal conditions. CP-1 and CP-2 were characterized by single-crystal X-ray diffraction, Fourier transform infrared spectroscopy, thermogravimetric analysis, elemental analysis, and powder X-ray diffraction analysis. Solid-state fluorescence analysis revealed an emission peak at 350 nm upon excitation at 225 and 290 nm. Fluorescence sensing tests showed that CP-1 was highly efficient, sensitive, and selective for detecting Cr_2_O_7_^2−^ at 225 and 290 nm, whereas I^−^ was only detected well at an excitation of 225 nm. CP-1 detected pesticides differently at excitation wavelengths of 225 and 290 nm; the highest quenching rates were for nitenpyram at 225 nm and imidacloprid at 290 nm. The quenching process may occur via the inner filter effect and fluorescence resonance energy transfer.

## 1. Introduction

With the rapid development of agriculture and industry, water pollution by inorganic species, such as Cr_2_O_7_^2−^ and I^−^, and organic compounds, such as pesticides, has become a serious problem [1]. Cr_2_O_7_^2−^ is a potent carcinogen that causes teratogenesis and may damage internal organs at acute doses [2]. Although I^−^ is essential for animal and plant life, and I^−^ deficiency can cause goiter and severely affect the normal development of children, excessive iodine intake can threaten health and can lead to hypermetabolic syndromes in the nervous, circulatory, digestive, and cardiovascular systems, as well as hyperexcitability [3,4]. Therefore, the effective detection of I^−^ has gradually attracted the interest of researchers. Pesticides are widely used in agriculture to prevent crop damage. However, the abuse of pesticides may leave excessive pesticide residues in plants, soil, and water [5]. The presence of these harmful substances in environmental waters threatens the environment and they may eventually harm our physical and mental health through the food chain [6]. Currently, these pollutants are analyzed by atomic absorption spectrometry, high-performance liquid chromatography, and gas chromatography [7,8]. However, these methods are time-consuming and require costly equipment; therefore, there is an urgent need to develop efficient, cost-effective, rapid assays for detecting these pollutants [9].

Coordination polymers (CPs) are an emerging class of organo-inorganic porous materials consisting of metal ions or clusters and organic linkers [10]. The diverse structures and functions of CP materials mean that they have potential applications in many fields, such as gas storage and separation [11], electronic sensing [12], electrocatalysis [13,14], photocatalysis [15], and magnetism [16,17]. In particular, fluorescence detection has attracted much attention because CPs can be used for detecting pollutants rapidly for real-time monitoring, and they have good reusability [18]. Due to their desirable properties, many fluorescent CPs have been designed and synthesized to detect various pollutants in water, including heavy metal ions [19], anions [20], pesticides [6,21], and volatile compounds [22], and to sense pH [23] and temperature [24].

Usually, the fluorescence emission site of CPs is an emissive metal center, consisting of a ligand and guest ion or molecule [25]. Many intrinsic and extrinsic factors, including the coordination properties of the metal centers, organic ligand structure, reaction temperature, solvents, pH, and molar ratio, can affect the formation of CPs greatly [26]. Suitable organic ligands are particularly important, and nitrogenous organic ligands and carboxylic acid ligands are key in tuning synergistic framework structures because their many coordination modes allow them to meet the geometric requirements of metal centers, leading to fascinating structural architectures [27]. Tuning the architecture of the CP with organic ligands is expected to be a good strategy for synthesizing fluorescent materials with good sensing performance. Several CPs based on Zn(II) and 1,3,5-benzenetricarboxylic acid (BTC) have been reported. It is well known that a basic understanding of the structures, their stability, and the electronic properties of Zn-BTC-CPs is crucial for their applications in various fields [28], mainly that of pesticide adsorption in real water samples [29], gas sensing [30], photocatalysis [31], antibacterial [32], and electrochemistry [33]. Until now, only a few experimental and theoretical studies have considered the M_3_(BTC)_2_ CPs family [34], and fluorescence-based detection of pesticides and ions in water has been less reported.

In the present work, two Zn(II) CPs, formed by the combination of BTC and nitrogen-containing ligands with Zn ion reported, namely: [Zn_2_(DIN)_2_(HBTC^2−^)_2_] (CP-1) and [Zn(DIN)(HBTC^2−^)] ACN·H_2_O (CP-2) (DIN = 1,4-di(imidazol-1-yl)naphthalene, H_3_BTC = 1,3,5-benzenetricarboxylic acid, and ACN = acetonitrile), were synthesized. CP-1 was used to study the fluorescence characteristics of different anions and pesticides in an aqueous solution. The results indicated that CP-1 functioned as a highly selective, reusable, and versatile sensor for detecting I^−^, Cr_2_O_7_^2−^, nitenpyram (NTP), and imidacloprid (IMI) under different excitation conditions by efficient fluorescence quenching detection.

## 2. Materials and Methods

### 2.1. Materials and Instruments

All the materials and solvents were obtained commercially and used without any further purification. ZnSO_4_·7H_2_O, benzene-1,3,5-tricarboxylic acid (H_3_BTC), and acetonitrile were supplied by Shanghai Aladdin Biochemical Technology Co., Ltd. (Shanghai, China); Bide Pharmatech Ltd. (Shanghai, China) supplied 2-[4-(2-Hydroxyethyl)-1-piperazinyl]ethanesulfonicacid (HEPES); 1,4-di(imidazole-1-yl)naphthalene (DIN) was synthesized following a reported method [35].

Powder X-ray diffraction (PXRD) data from all samples were collected from Bruker-avance X-ray diffractometer (Bruker Corporation, Karlsruhe, Germany), in which the X-ray tube was a Cu-target with the range of 5–50° at the rate of 0.2 °/s. The TG curve was obtained on METTLER TOLEDO 1600TH thermal analyzer (Mettler-Toledo International Inc., Zurich, Switzerland) which was operated under an N_2_ atmosphere and at a heating rate of 10 °C/min over the temperature ranging from room temperature (r.t.) to 800 °C in a flowing nitrogen atmosphere of 10 mL/min using platinum crucibles. Agilent Cary630 spectrophotometer (Agilent Technologies Co., Ltd., Santa Clara City, CA, USA) was used for the recording of Fourier transform infrared (FT-IR) in the range of 4000 to 500 cm^−1^. The UV-Vis absorption spectra were recorded on the Varian Cary UV50 spectrophotometer (Varian Medical Systems, Inc., Palo Alto, CA, USA). The fluorescence excitation/emission spectra of the samples were studied at r.t. using an Agilent Cary Eclips fluorescence spectrophotometer (Agilent Technologies Co. Ltd., Santa Clara City, CA, USA). Using the Mercury software version 4.0 (Cambridge Crystallographic Data Centre, Cambridge, UK), the simulated X-ray diffraction patterns were generated from properly treated Cif files of the related complex crystals. Elemental analyses (C, H, and N) were performed on Perkin-Elmer 240 CHN elemental analyzer (Perkin-Elmer inc., Waltham, MA, USA).

### 2.2. Single-Crystal X-ray Diffraction Analysis

Single crystals suitable for X-ray diffraction analysis of CP-1 and CP-2 were placed on the tip of the goniometer head on Bruker APEX-II CCD diffractometer and were kept at 150.0(1) K. X-ray data collection was obtained under graphite monochromated Mo-K*α* radiation (*λ* = 0.71073 Å). The Olex2 (OlexSys Ltd., Durham, UK) [36] software was used to solve the structures by Direct Methods with the SIR 2004 structure solution program [37]. The SAINT program [38] was used for obtaining integration and scaling of intensity data. Data were corrected for the effects of absorption using SADABS [39,40]. The ShelXL [41] was used for refining the structures with a refinement package using Least Squares minimization. All non-hydrogen atoms were refined anisotropically. The hydrogen atoms in the riding mode [36,42] and isotropic temperature factors fixed at 1.2 times U(eq) of the parent atoms. A summary of the crystallographic data of refinements is given in Table S1 (Supplementary Materials). CCDC numbers for CP-1 and CP-2 deposited at Cambridge Crystallographic Data Center are 2,133,163 and 2,133,166.

### 2.3. Synthesis of CP-1 and CP-2

To a Teflon-lined stainless-steel autoclave (25 mL), ZnSO_4_·7H_2_O (1 eq., 0.0086 g, 0.030 mmol), DIN (1 eq., 0.0078 g, 0.030 mmol), BTC (1 eq., 0.0063 g, 0.030 mmol) and the solvents were added and sealed. After being stirred and ultrasound for 10 min, the mixture was maintained at 80 °C for 72 h. After the reaction was then slowly allowed to r.t., the crystallized solid material was obtained. The white crystals were filtered and washed with deionized water and were dried in the open air at r.t. for two days. Crystals that were suitable for X-ray diffraction analysis were obtained from the synthesis process and analyzed without further treatment.

CP-1: *N*,*N*-dimethylacetamide/H_2_O (*v*/*v* = 1:1, 5 mL) (0.021 g, yield: 65%). Elemental analysis for C_50_H_32_N_8_O_12_Zn_2_ (wt %) Calcd.: C, 56.25%; H, 3.02%; N, 10.50%; found: C, 56.16%, H, 2.98%, N, 10.05%; IR (neat, *ν*/cm^−1^): 3050(w), 1705(s), 1626(s), 1576(m), 1421(m), 1328(m), 1248(s), 1099(s), 1082(s), 1038(s), 944(m), 851(m), 754(s), 724(s), 680(m), 656(s), and 562(m). s = strong; m = moderate; w = weak.

CP-2: ACN and H_2_O (*v*/*v* = 1:1, 5 mL) (0.010 g, yield: 57%). Elemental analysis for C_27_H_21_N_5_O_7_Zn (wt %) Calcd.: C, 54.70%; H, 3.57%; N, 11.80%; found: C, 54.56%, H, 3.51%, N, 11.75%; IR (neat, *ν*/cm^−1^): 3123(w), 2221(w), 1697(m), 1578(m), 1436(m), 1347(s), 1244(m), 1082(s), 950(m), 846(m), 754(s), 728(s), 656(s), and 514(m).

### 2.4. Analyte Quenching Test

Before the fluorescence sensing test, CP-1 was powdered and suspended in buffer solution (HEPES) at a concentration of 2 mg/mL by ultrasonication for 30 min. Aqueous analyte solutions with concentrations of 2 mM (ions) and 0.2 mM (pesticides) were prepared. The analytes were as follows. Ions: MCl_1–3_ (M = Cd^2+^, Ba^2+^, Ca^2+^, Al^3+^, Cr^3+^, Co^2+^, In^3+^, Mn^2+^, K^+^, Mg^2+^, Ni^2+^, Na^+^, Pb^2+^, Zn^2+^) and K_1–2_X (X = Br^−^, Ac^−^, B_4_O_7_^2−^, ClO_4_^−^, ClO_3_^−^, Cl^−^, CO_3_^2−^, Cr_2_O_7_^2−^, F^2−^, HPO_4_^2−^, H_2_PO_4_^−^, I^−^, NO_3_^−^, SCN^−^, SO_3_^2−^, and SO_4_^2−^). Pesticides: carbendazim (CAR), dipterex (DIP), 2,4-dichlorophenoxyacetic acid (2,4-D), imidacloprid (IMI) imazalil (IMZ), glyphosate (GLY), nitenpyram (NTP), pentachloronitrobenzene (PCNB), metamitron (MMT), thiophanate-methyl (TPM), and tetrachloroisophthalonitrile (chlorothalonil; TPN). Fluorescence sensing was examined by measuring the fluorescence spectra at excitation wavelengths (*λ*_ex_) of 225 and 290 nm of mixtures of equal amounts of the CP suspension and analyte solutions, and of the CP suspension alone.

### 2.5. Fluorescence Kinetic Titration

Titration was performed by adding finely ground CP-1 (4 mg) to distilled water (40 mL) and ultrasonicating the mixture for 0.5 h. Solutions of the ions (20 mM) and the pesticides (5 mM) were prepared. In each sensing experiment, the analyte solution (2–10 μL) was added to the aqueous dispersion of CP-1 (4 mL). The fluorescent spectra of the mixtures were recorded.

## 3. Results and Discussion

### 3.1. Structures of CP-1 and CP-2

**Figure 1:**
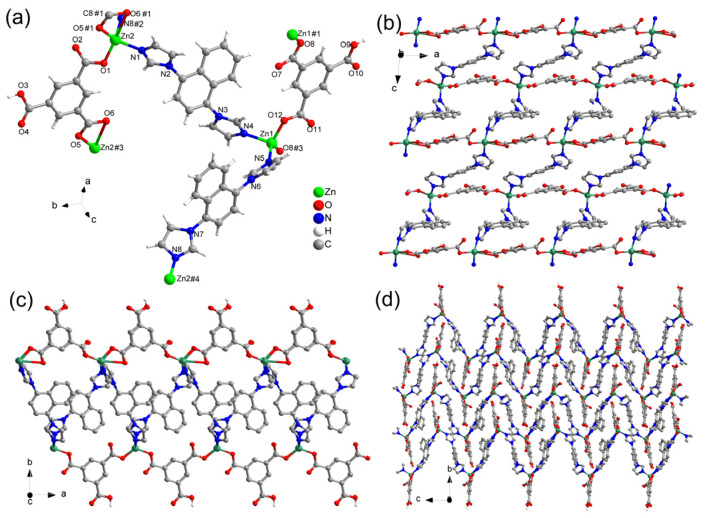
(**a**) Coordination environment of Zn(II) ions, HBTC^2−^, and DIN ligands in CP-1; (**b**) view of the 2D framework along the *b*-axis; (**c**) view of the 2D framework along the *c*-axis; (**d**) view of the 2D frameworks along the *a*-axis.

The single-crystal X-ray diffraction analysis revealed that CP-1 and CP-2 crystallize in the monoclinic P2_1_/n (14) space group. The asymmetric unit of CP-1 contains two crystallographically independent Zn(II) centers (Zn1 and Zn2), two incompletely deprotonated HBTC^2−^ ligands, and two DIN ligands (Figure 1). Similarly, the asymmetric unit of CP-2 consists of one incompletely deprotonated HBTC^2−^ ligand and one DIN ligand, as well as one lattice ACN and one lattice water molecule.

In CP-1, the Zn1 center is four-coordinated (Figure 1a) by two O atoms from two HBTC^2−^ ligands and two N atoms from two DIN linkers. The Zn2 center is five-coordinated by three O atoms from two HBTC^2−^ ligands and two N atoms from two different DIN linkers. The HBTC^2−^ and DIN ligands function as bridging linkers that connect the two Zn(II) centers. The Zn(II) centers and HBTC^2−^ alternate to form a chain along the *a*-axis. The neighboring chains are connected by DIN ligands via the Zn(II) cores to generate a two-dimensional (2D) layer on the *aoc* plane (Figure 1b,c). The stacked layers of CP-1 in the direction of the *b*-axis are connected by additional strong and weak hydrogen bonds and *π*-*π* interactions of the nearby rings. The hydrogen bonds include O3–H···O7#1 (carboxylate) and O9–H···O6#2 (carboxylate) with donor–H···acceptor distances of 2.540(3) and 2.557(3) Å, respectively. There are *π*-*π* interactions between the benzene (C11–C16) and (C42–C46) rings with a centroid–centroid (Cg···Cg#3) distance of 3.771(2) Å. These bonds together with additional weak hydrogen bonds and the *π*-*π* interactions of the rings allows CP-1 to form a three-dimensional supramolecular framework (Figure 1d) [symmetry code: #1: −1/2 + *x*, 3/2 − *y*, −1/2 + *z*; #2: 1 − *x*, 1 − *y*, 1 − *z*; #3: 1 − *x*, 1 − *y*, 2 − *z*].

**Figure 2:**
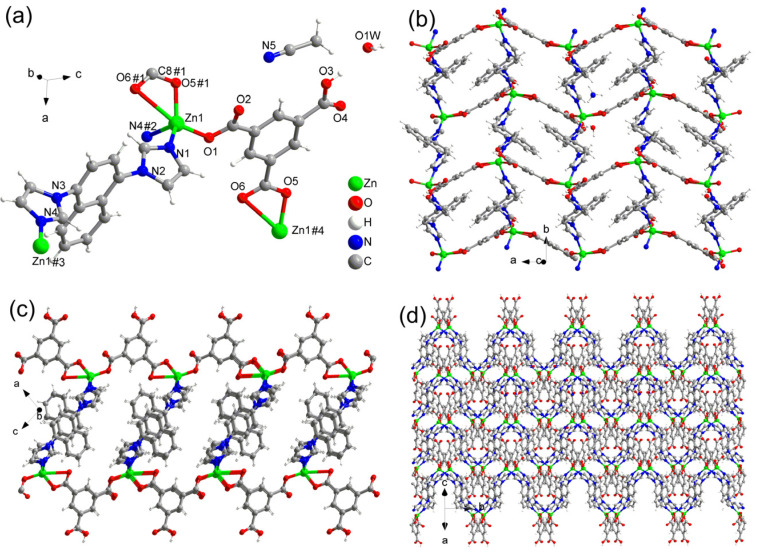
(**a**) Coordination environment of Zn(II) ion, HBTC^2−^, and DIN ligands in CP-2; (**b**) view of the 2D framework along the *c*-axis; (**c**) view of the 2D framework along the *b*-axis; (**d**) view of the 2D framework along the *aoc* vector.

The coordination modes of the Zn(II) ions and the ligands in CP-2 are similar to those in CP-1. The architecture of CP-2 is also a 2D network (Figure 2a–c), which forms a three-dimensional supramolecule (Figure 2d) via strong and weak hydrogen bonds and weak *π*-*π* interactions of the rings in the direction of the *aoc* vector. The lattice water molecules bridge three sheets via three hydrogen bonds of O3–H···O1W (water), O1W–H···O2#1 (carboxylate), and O1W–H···O6#2 (carboxylate) with bond lengths of 2.628(3), 2.745(3), and 2.769(3) Å, respectively.

### 3.2. Fourier Transform Infrared Spectroscopy, Thermogravimetric Analysis, and Powder X-ray Diffraction of CP-1 and CP-2

To characterize CP-1 and CP-2 further, Fourier transform infrared (FT-IR) spectroscopy, thermogravimetric analysis (TGA) and powder X-ray diffraction (PXRD) were performed. To determine the phase purities of CP-1 and CP-2, PXRD was performed on the as-synthesized samples. Figure S1 (Supplementary Materials) shows that the experimental PXRD patterns were consistent with the simulated ones, confirming that the materials consisted of a single phase.

In the FT-IR spectrum, the carboxyl group (-COOH) O-H stretching vibration (*V*_O-H_; 1700–1680 cm^−1^), the carbonyl group (-C=O) stretching vibration (*V*_C=O_; 3300–2500 cm^−1^), and the O-H out of plane bending vibration (*δ*_O-H_; 950–890 cm^−1^) were the three main characteristic frequencies [28,43]. For CP-1, Figure S2a (Supplementary Materials) shows that the characteristic peaks associated with *V*_O-H_ remained, and new peak appeared at 1626(s), and two sets of symmetric peaks appeared that were attributed to the asymmetric stretching vibration (1576 cm^−1^) and symmetric stretching vibration (1421 cm^−1^). The characteristic *V*_C=O_ peaks did not disappear completely, and CP-1 still had a small absorption at 3100 cm^−1^, which may be due to water molecules. Characteristic *δ*_O-H_ peaks at 944 cm^−1^ also did not disappear. The FT-IR spectrum of CP-2 (Figure S3b, Supplementary Materials) shows that the peaks related to *V*_O-H_ peaks appeared at 1697 cm^−1^ and asymmetric and symmetric stretching vibrations were observed at 1578 and 1436 cm^−1^, respectively. As with CP-1, the characteristic peaks of *V*_C=O_ and *δ*_O-H_ did not disappear completely, and CP-2 still showed absorptions at 3123 and 950 cm^−1^. In addition, the FT-IR spectrum of CP-2 contained a distinct peak at 2221 cm^−1^, which is characteristic of the nitrile group (-C≡N) in ACN (Figure S3b, Supplementary Materials), although the peak is shifted by 44 cm^−1^ compared with that of gas-phase ACN (2265 cm^−1^). The solid-state structural data of CP-1 and CP-2 were consistent with the FT-IR data.

TGA spectra of the CPs are shown in Figure S3 (Supplementary Materials). For CP-1, the coordination framework exhibited no obvious weight loss up to 400 °C, followed by fast weight loss, indicating that the CP began to decompose rapidly. A weight loss of 10.7% (calcd 10.0%) was observed in the TGA curve of CP-2 from room temperature to 145 °C, which was attributed to the removal of lattice ACN and water molecules. As the temperature increased further, a sharp weight loss occurred above 400 °C, marking the beginning of the decomposition of the framework. Furthermore, to evaluate the stability of CP-1. CP-1 and CP-2 were placed in water (100 or 200 °C) or in air (50 or 100 °C) for 24 h, and subsequently the PXRD of CP-1 and CP-2 were tested under each condition. The results are shown that the PXRD test spectra of the samples agree with the simulated spectra. This result implies that the material can remain intact after the stability test (Figure S4, Supplementary Materials). The stability in the buffer solution (HEPES) was also tested, and it was found that the PXRD of the material remained stable before and after adding the buffer solution. CP-1 and CP-2 are considered to be a material with good stability properties.

### 3.3. Fluorescence of the CPs

The structures of *d*^10^ metals are good luminescent centers and are often used to construct potential fluorescent CPs. Therefore, the fluorescence emissions of CP-1 and CP-2 in the buffer solution were evaluated. For both CPs, a broad emission peak was observed with a maximum wavelength (*λ*_max_) at 350 nm (Figure S5, Supplementary Materials) at *λ*_ex_ of 225 and 290 nm. The excitation and emission peaks of the CPs resembled those of the free DIN ligand, indicating that both materials exhibited similar ligand-based fluorescence. Consequently, the following sections discuss the evaluation of CP-1 as a fluorescence sensor.

### 3.4. Fluorescence Sensing Behavior of CP-1 for Ions in Buffer Solution

**Figure 3:**
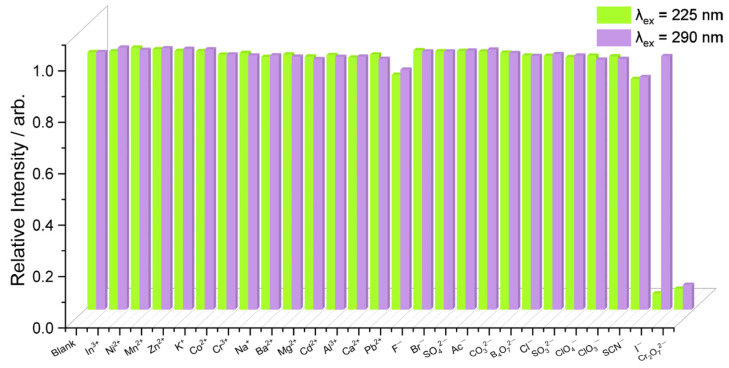
Fluorescence intensity of CP-1 dispersed in an aqueous solution (pH = 7.0) after the addition of various ions.

The pH sensing experiments were performed before the sensing ion experiments, as demonstrated in Figure S6 (Supplementary Materials). CP-1 was evaluated as a fluorescence sensor for various ions (Al^3+^, Ba^2+^, Ca^2+^, Cd^2+^, Cr^3+^, Co^2+^, In^3+^, Mn^2+^, K^+^, Mg^2+^, Ni^2+^, Na^+^, Pb^2+^, Zn^2+^, Ac^−^, Br^−^, B_4_O_7_^2−^, Cl^−^, ClO_3_^−^, ClO_4_^−^, CO_3_^2−^, Cr_2_O_7_^2−^, F^−^, I^−^, SCN^−^, SO_3_^2−^, and SO_4_^2−^). Each ion solution (0.2 mM) was added to a suspension of CP-1 (0.2 mg/mL) in a neutral buffer solution (H_2_PO_4_^−^ and HPO_4_^2−^) and the fluorescence emission spectrum of the sensor was recorded. The addition of ions altered the fluorescence intensity of the sensor (Figure 3). The strongest quenching effect was observed for I^−^ at *λ*_ex_ of 225 nm and for Cr_2_O_7_^2−^ at *λ*_ex_ of 225 and 290 nm. In addition, Pb^2+^ and SCN^−^ decreased fluorescence intensity by about 10% compared with CP-1 alone, and other anions showed slight or negligible effects on the emission intensity of CP-1.

In addition to the fluorescence sensing that was conducted at a single sensor concentration, kinetic titration experiments were performed by gradually increasing concentrations of I^−^ or Cr_2_O_7_^2−^ in a neutral buffer suspension of the sensor. The fluorescence intensities as a function of the concentration of the quenchers were evaluated by the Stern–Volmer (SV) equation, I_0_/I = 1 + *K*_sv_ [M], where *I*_0_ is the sensor fluorescence intensity, *I* is the sensor fluorescence intensity after the addition of a quencher, and [M] is the molar concentration of the quencher [44]. Figure S7 (Supplementary Materials) shows that at *λ*_ex_ of 225 nm, I_0_/I—1 versus [I^−^] was well fitted by the SV equation (K_sv_ = 2.16 × 10^4^ M^−1^) at low quencher concentrations, whereas at higher quencher concentrations, the *K*_sv_ values deviated from the linear fitting. The results were similar for the plots of I_0_/I—1 vevrsus [Cr_2_O_7_^2−^] at *λ*_ex_ of 225 and 290 nm, and the calculated *K*_sv_ values were 1.08 × 10^4^ (Figure S8, Supplementary Materials) and 1.21 × 10^4^ M^−1^ M^−1^ (Figure S9, Supplementary Materials), respectively, at low Cr_2_O_7_^2−^ concentrations. In addition, the limit of detection (LOD) was evaluated based on the equation LOD = 3*σ*/*K*_sv_ [45,46], where *σ* is the standard deviation calculated from 10 repeat luminescence values of the original suspension. By using the *K*_sv_ values, the LODs were calculated as 0.33 μM (*λ*_ex_ = 225 nm) for I^−^ and 0.50 μM (*λ*_ex_ = 225 nm) and 0.44 μM (*λ*_ex_ = 290 nm) for Cr_2_O_7_^2−^.

### 3.5. Competitive Fluorescence Quenching

**Figure 4:**
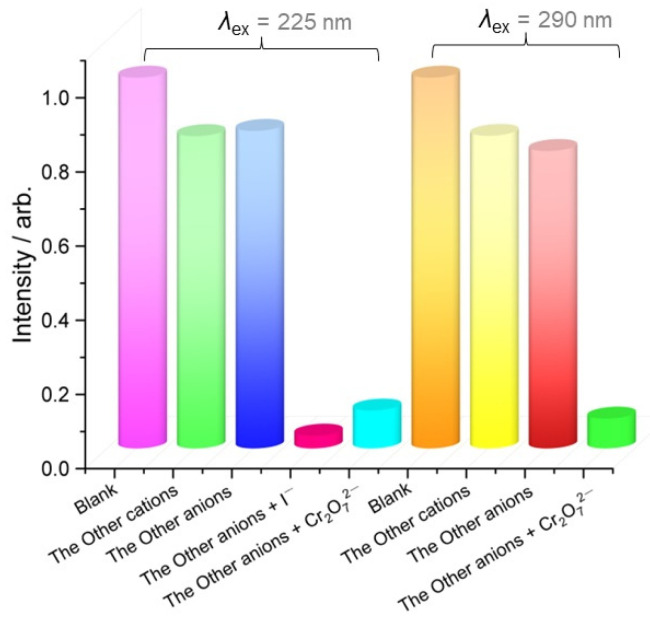
Competitive fluorescence quenching experiments for selective recognition of I^−^ and Cr_2_O_7_^2−^ by CP-1.

The ion selectivities of CP-1 for I^−^ and Cr_2_O_7_^2−^ were tested in the presence of the other cations and anions. Figure 4 shows that the fluorescence intensity of CP-1 decreased from 80% to 20% in the presence of the other cations or anions (*λ*_ex_ = 225 or 290 nm) compared with CP-1 alone. Substantial quenching occurred when I^−^ (*λ*_ex_ = 225 nm) and Cr_2_O_7_^2−^ (*λ*_ex_ = 225 and 290 nm) were added to CP-1. Therefore, CP-1 exhibited good selectivity toward I^−^ and Cr_2_O_7_^2−^ in the presence of other cations or anions.

### 3.6. Fluorescence Sensing Behavior of CP-1 towards Pesticides

**Figure 5:**
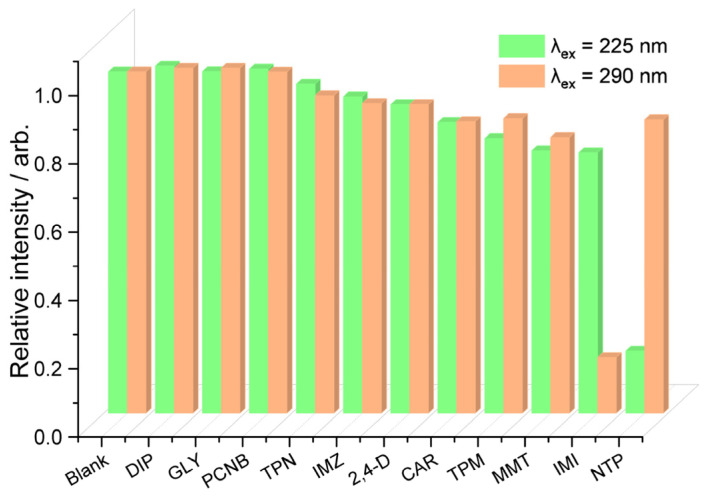
Fluorescence intensity of CP-1 dispersed in neutral aqueous solutions after the addition of various pesticides.

The fluorescence sensing of CP-1 toward common pesticides was tested by adding pesticide solution (0.2 mM) to a suspension of the sensor (0.2 mg/L) in a neutral buffer solution. Figure 5 shows that DIP, GLY, and PCNB had little effect on the fluorescence, whereas the other compounds caused fluorescence quenching to some degree. At *λ*_ex_ of 225 nm, the fluorescence quenching was in the order TPN > IMZ > 2,4-D > CAR > TPM > MMT > IMI and NTP. At *λ*_ex_ of 290 nm, the fluorescence quenching was in the order 2,4-D < TPN < IMZ < TPM < MMT < NTP < IMI. Therefore, NTP and IMI at *λ*_ex_ of 225 and 290 nm, respectively, induced the largest fluorescence quenching. Then, the titration experiments were performed on NTP and IMI at *λ*_ex_ of 225 and 290 nm, respectively. The fluorescence intensity of CP-1 decreased gradually with increasing NTP and IMI concentration. The curves were linear at low analyte concentrations, but non-linear at higher analyte concentrations. In the linear range of the curves, for NTP, *K*_sv_ was 3.06 × 10^4^ M^−1^ and LOD was 0.28 μM (*λ*_ex_ = 225 nm) (Figure S10, Supplementary Materials), and for IMI, *K*_sv_ was 2.91 × 10^4^ M^−1^ and LOD was 0.25 μM (*λ*_ex_ = 290 nm) (Figure S11, Supplementary Materials).

### 3.7. Reusability of CP-1

**Figure 6:**
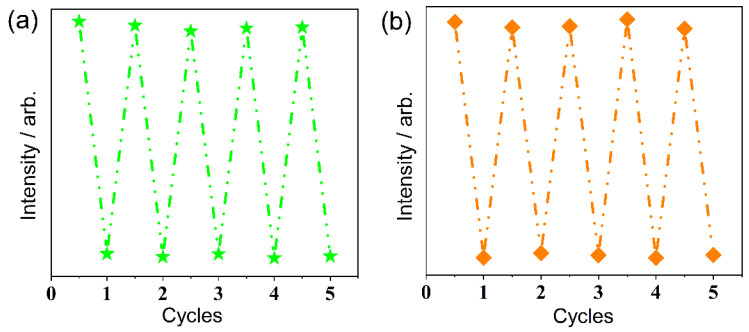
Reversibility of CP-1 for (**a**) NTP (*λ*_ex_ = 225 nm); (**b**) IMI (*λ*_ex_ = 290 nm).

Reusability is an important practical feature of fluorescent probes. Thus, the reusability of CP-1 for fluorescence sensing was investigated. CP-1 was regenerated several times by simply centrifuging the suspension followed by repeated washing with water. As shown in Figure 6, the initial intensity after five cycles was almost unchanged, indicating excellent reusability.

### 3.8. Possible Sensing Mechanism

The mechanism of the highly sensitive recognition of I^−^, Cr_2_O_7_^2−^, NTP, and IMI was investigated. First, PXRD and FT-IR were performed on CP-1 before and after analyte sensing. The results in Figure S8 (Supplementary Materials) show that the peaks in the PXRD and FT-IR spectra after sensing were consistent with those for as-synthesized CP-1. Therefore, the structure of CP-1 remained intact after sensing and I^−^, Cr_2_O_7_^2−^, NTP, and IMI did not induce fluorescence quenching by destroying CP-1. In addition, in many cases [47,48], photo-induced electron transfer (PET) [49] may involve in the fluorescence quenching process, due to the fact that the fluorescence of CP-1 is based on the DIN ligand. The energy levels of the DIN ligand were employed to represent the CP-1 to compare with that of the NTP and IMI by using the support of density functional theory (DFT) calculations [50,51,52,53,54,55,56,57,58]. The results are shown in Figure S12 (Supplementary Materials). The energy level of the lowest unoccupied molecular orbital (LUMO) of DIN (−2.57 eV) is lower than that of the LUMO of NTP (−2.11 eV) and IMI (−2.27 eV). Therefore, no excitation electron is expected to transfer from the DIN ligand to the LUMOs of the analytes, ruling out the involvement of the PET during the fluorescence quenching.

**Figure 7:**
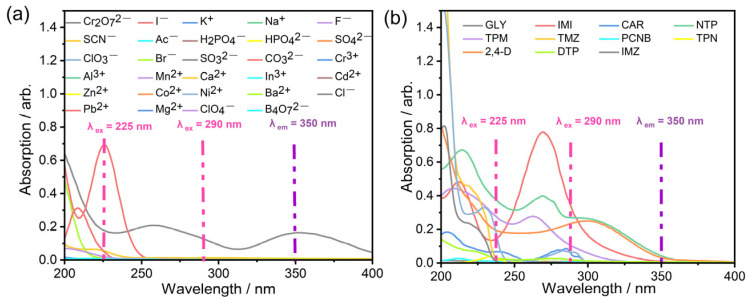
UV-Vis absorption spectra of different (**a**) ions; (**b**) pesticides.

**Figure 8:**
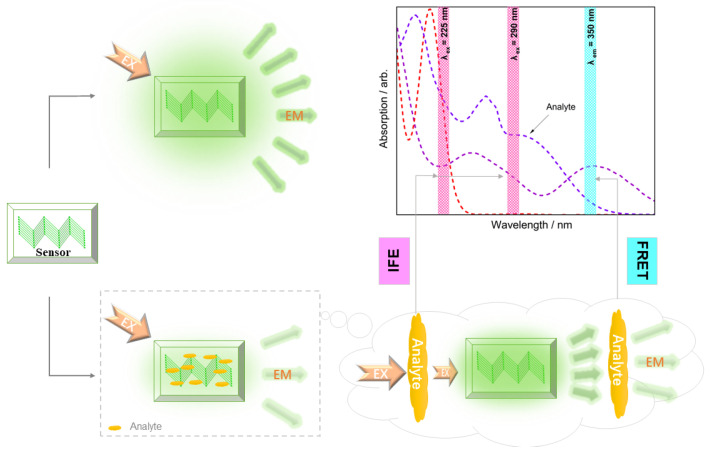
Schematic of the quenching mechanisms for the CPs and their relationship with the observed UV-Vis absorption spectrum.

In addition, the UV absorption spectra of the ions (Figure 7a) and the pesticides (Figure 7b) were then evaluated together with the fluorescence of CP-1. At 225 nm, there was a strong overlap between the excitation of the CP and the UV-Vis absorption of both I^−^ and Cr_2_O_7_^2−^, and substantial fluorescence quenching caused by the two ions was observed. Therefore, the inner filter effect (IFE) [59] mechanism was involved in the fluorescence quenching process. However, at 290 nm, there was no UV-Vis absorption by I^−^, and no obvious fluorescence quenching was observed; thus, the IFE mechanism dominated the quenching by I^−^. In addition to the overlap at 225 and 290 nm, there was a superposition at 350 nm of the Cr_2_O_7_^2−^ absorption and the fluorescence emission of CP-1, suggesting that fluorescence resonance energy transfer (FRET) [60] was also involved in the fluorescence quenching. The UV absorption spectra of the pesticides (Figure 7b) at *λ*_ex_ of 225 and 290 nm and the induced fluorescence quenching were evaluated. The stronger the UV-Vis absorption of the analytes was, the stronger the fluorescence quenching of the sensor, demonstrating that the IFE mechanism dominated the fluorescence quenching of CP-1 during the detection of the pesticides. The mechanisms are summarized in Figure 8.

## 4. Conclusions

Two reusable, sensitive, and versatile sensors were synthesized from DIN, H_3_BTC, and ZnSO_4_ under solvothermal conditions. The CPs were characterized by single-crystal X-ray diffraction, FT-IR, TGA, elemental analysis, and PXRD. Structural analysis showed that both CPs had 2D architectures. Both CPs also demonstrated similar fluorescence properties of a doublet excitation peak with wavelengths at 225 and 290 nm and similar intensities and a singlet emission peak at 350 nm. The excitation wavelengths exhibited high efficiency, selectivity, and sensitivity for different anions and pesticides in fluorescence sensing experiments, particularly for the anions I^−^ (*λ*_ex_ = 225 nm) and Cr_2_O_7_^2−^ (*λ*_ex_ = 225 and 290 nm) and for the pesticides NTP (*λ*_ex_ = 225) and IMI (*λ*_ex_ = 290 nm). In addition, the possible quenching mechanisms were identified as IFE and FRET. This study provides a feasible approach for designing MOF sensors to cope with metal ions, antibiotics, and pesticides in water. It is anticipated that these fluorescent MOFs may have great potential for contaminant sensing and contaminant separation.

## Data Availability

Data are available from the corresponding author upon reasonable request.

## Supplementary Materials

The following supporting information can be downloaded at: https://www.mdpi.com/article/10.3390/polym15112570/s1, Figure S1: PXRD patterns of simulated and as-synthesized CP-1 and CP-2, Figure S2: FT–IR spectra of CP-1 and CP-2, Figure S3: TGA spectra of CP-1 and CP-2, Figure S4: (a) PXRD patterns of CP-1 and CP-2 in air at 50 °C or 100 °C; in water at 100 °C or 200 °C, Figure S5: Fluorescence spectra of CP-1 (a) and CP-2 (b) in aqueous suspension at r.t.: excitation (insert) and emission, Figure S6: pH-dependent fluorescence and linear variation (insert); (a) excitation of 225 nm (b) excitation of 290 nm, Figure S7: Fluorescence intensities of CP-1 (a) dispersed in different concentrations of I^−^; the plot of I_0_/I—1 of CP-1; (b) vs. concentration of I^−^ in aqueous solution (pH = 7.0) at 225 nm (Insert: The plot of I_0_/I—1 of the CPs with the concentration over a I^−^ concentration range of 0–0.1 mM in aqueous solution), Figure S8: Fluorescence intensities of CP-1 (a) dispersed in different concentrations of Cr_2_O_7_^2−^; the plot of I_0_/I—1 of CP-1; (b) vs. concentration of Cr_2_O_7_^2−^ in aqueous solution (pH = 7.0) at 225 nm (Insert: The plot of I_0_/I—1 of the CPs with the concentration over a Cr_2_O_7_^2−^ concentration range of 0–0.09 mM in aqueous solution), Figure S9: Fluorescence intensities of CP-1 (a) dispersed in different concentrations of Cr_2_O_7_^2−^; the plot of I_0_/I—1 of CP-1 (b) vs. concentration of Cr_2_O_7_^2−^ in aqueous solution (pH = 7.0) at 290 nm (Insert: The plot of I_0_/I—1 of the CPs with the concentration over a Cr_2_O_7_^2−^ concentration range of 0–0.09 mM in aqueous solution), Figure S10: Fluorescence intensities of CP-1 (a) dispersed in different concentrations of NTP; the plot of I_0_/I—1 of CP-1 (b) vs. concentration of NTP in aqueous solution (pH = 7.0) at 225 nm (Insert: The plot of I_0_/I—1 of the CPs with the concentration over a NTP concentration range of 0–0.08 mM in aqueous solution), Figure S11: Fluorescence intensities of CP-1 (a) dispersed in different concentrations of IMI; the plot of I_0_/I—1 of CP-1 (b) vs. concentration of IMI in aqueous solution (pH = 7.0) at 290 nm (Insert: The plot of I_0_/I—1 of the CPs with the concentration over a IMI concentration range of 0–0.08 mM in aqueous solution), Figure S12: The calculated HOMO and LUMO energy levels of DIN ligand, IMI and NTP, Table S1: Crystal data and structure refinement details for CP-1 and CP-2, Table S2: The selected bond lengths [Å] and angles [°] for CP-1, Table S3: The selected bond lengths [Å] and angles [°] for CP-2, Table S4: *K*_sv_ and LOD values of CP-1 for ions detection. Table S5: Chemical structures of the selected pesticides. Table S6: Ksv and LOD values for recently reported Zn-MOF-based luminescence probes for sensing of NTP and IMI.
