# Supplementary material for: Zn(II)-Based Mixed-Ligand-Bearing Coordination Polymers as Multi-Responsive Fluorescent Sensors for Detecting Dichromate, Iodide, Nitenpyram, and Imidacloprid
Source: Polymers (Basel). 2023 Jun 2;15(11):2570. doi: 10.3390/polym15112570 (PMC10255766; doi:10.3390/polym15112570)
Supplement: Supplementary file 1 [file polymers-15-02570-s001.zip › polymers-2359483-supplementary.pdf]

polymers

MDPI

Supplementary Information

Zn(II)-Based Mixed-Ligand-Bearing Coordination Polymers as Multi-Responsive Fluorescent Sensors for Detecting Dichromate, Iodide, Nitenpyram, and Imidacloprid

Dan Wang [1], Lin-Huan Du [1], Long Li [1], Yu-Meng Wei [1], Tao Wang [1], Jun Cheng [1], Bin Du [2], Yi Jia [3] and Bao-Yi Yu [1,*]

1 Key Laboratory of Urban Agriculture in North China, Ministry of Agriculture and Rural Affairs, Beijing University of Agriculture, Beijing 102206, China; 202130123127@bua.edu.cn (D.W.); 202120151031@bua.edu.cn (L.-H.D.); 202020152122@bua.edu.cn (Y.-M.W.); 202220151161@bua.edu.cn (T.W.); chengjun@bua.edu.cn (J.C.)
2 Beijing Key Laboratory of Agricultural Product Detection and Control of Spoilage Organisms and Pesticide Residue, Faculty of Food Science and Engineering, Beijing University of Agriculture, Beijing 102206, China; bindu80@bua.edu.cn
3 Beijing National Laboratory for Molecular Sciences, CAS Key Lab of Colloid, Interface and Chemical Thermodynamics, Institute of Chemistry, Chinese Academy of Sciences, Beijing 100190, China; jiayi@iccas.ac.cn
* Correspondence: baoyi.yu@bua.edu.cn; Tel.: +86-13511066021

Table S1. Crystal data and structure refinement details for CP-1 and CP-2.

CDC No.	2133163	2133166
Formula	$C_{50}H_{32}N_8O_{12}Zn_2$	$C_{27}H_{21}N_5O_7Zn$
Formula weight	1067.57	592.86
Temperature/K	150.0(1)	150.0(1)
Crystal system	monoclinic	monoclinic
Space group	$P2_1/n$	$P2_1/n$
a / Å	9.5028(7)	13.143(1)
b / Å	35.052(3)	14.443(1)
c / Å	13.407(1)	14.014(1)
α / °	90	90
β / °	95.583(3)	97.600(4)
γ / °	90	90
V / Å^3	4444.4(6)	2636.8(5)
Z	4	4
ρ_{calc}[g/cm^3]	1.595	1.493
μ / mm^{-1}	1.158	0.987
F(000)	2176.0	1216.0
Crystal size / mm^3	0.23 × 0.21 × 0.2	0.27 × 0.21 × 0.19
Radiation	MoKα (λ = 0.71073)	MoKα (λ = 0.71073)
2θ range for data collection / °	4.424 to 52.892	3.994 to 52.776
Index ranges	-11 ⩽ h ⩽ 11, -43 ⩽ k ⩽ 43, -14 ⩽ l ⩽ 16	-16 ⩽ h ⩽ 16, -18 ⩽ k ⩽ 17, -17 ⩽ l ⩽ 17
Reflections collected	34195	25800
Independent reflections	9040 [R_{int} = 0.0677, R_{sigma} = 0.0647]	5398 [R_{int} = 0.0857, R_{sigma} = 0.0695]
Data/restraints/parameters	9040/0/651	5398/0/366
Goodness-of-fit on F^2	1.038	1.042

Final R indexes [$I \geqslant 2\sigma(I)$][a]	R_1 = 0.0432, wR_2 = 0.0843	R_1 = 0.0431, wR_2 = 0.0855
Final R indexes [all data][b]	R_1 = 0.0762, wR_2 = 0.0997	R_1 = 0.0757, wR_2 = 0.0981
Largest diff. peak/hole / e Å^{-3}	1.21/-0.43	0.40/-0.41

[a] $R_1 = \Sigma_1|F_0|-|F_C|/\Sigma_1|F_0|$; [b] $wR_2 = [\Sigma[w(F_0^2-F_C^2)^2]/\Sigma[(F_0^2)^2]]^{1/2}$.

Table S2. The selected bond lengths [Å] and angles [°] for CP-1.

Zn1-O8$^{\#1}$	1.939(2)	N1-C20	1.379(4)	C6-C8	1.496(4)	C13-C12	1.391(4)
Zn1-O12	1.934(2)	O10-C18	1.206(4)	C6-C5	1.392(4)	C13-C18	1.484(4)
Zn1-N4	2.032(3)	O7C17	1.226(4)	C10-C11	1.509(4)	C27-C28	1.409(4)
Zn1-N5	2.026(3)	N8-C48	1.320(4)	C20-C21	1.345(4)	C27-C22	1.417(4)
Zn2-O5$^{\#2}$	1.964(2)	N8-C49	1.395(5)	C16-C11	1.390(4)	C24-C23	1.408(4)
Zn2-O1	1.942(2)	N2-C19	1.343(4)	C16-C15	1.392(4)	C22-C23	1.356(5)
Zn2-N1	2.016(3)	N2-C22	1.443(4)	C4-C5	1.391(4)	C30-C31	1.361(5)
Zn2-N8$^{\#3}$	2.043(3)	N2-C21	1.381(4)	C4-C3	1.387(4)	C42-C41	1.417(5)
O8-C17	1.270(4)	N3-C32	1.342(4)	C4-C9	1.490(4)	C42-C43	1.429(5)
O5-C8	1.267(4)	N3-C25	1.445(4)	C1-C2	1.502(4)	C42-C47	1.420(5)
O4-C9	1.208(4)	N3-C34	1.373(4)	C14-C15	1.388(4)	C38-C43	1.425(5)
O12-C10	1.285(4)	N5-C35	1.321(4)	C14-C13	1.395(4)	C38-C39	1.356(5)
O1-C1	1.283(4)	N5-C36	1.372(5)	C25-C26	1.418(4)	C49-C50	1.352(5)
O2-C1	1.230(4)	N7-C48	1.353(4)	C25-C24	1.365(4)	C41-C40	1.357(5)
O11-C10	1.227(4)	N7-C41	1.446(4)	C11-C12	1.388(4)	C43-C44	1.416(5)
O6-C8	1.243(4)	N7-C50	1.384(4)	C29-C28	1.362(4)	C36-C37	1.352(5)
N4-C32	1.315(4)	N6-C35	1.339(4)	C29-C30	1.411(5)	C33-C34	1.354(5)
N4-C33	1.377(4)	N6-C38	1.449(4)	C2-C3	1.389(4)	C40-C39	1.407(5)
O9-C18	1.314(4)	N6-C37	1.375(5)	C17-C15	1.501(4)	C44-C45	1.368(5)
O3-C9	1.325(4)	C7-C6	1.391(4)	C26-C27	1.438(4)	C46-C47	1.356(5)
N1-C19	1.325(4)	C7-C2	1.388(4)	C26-C31	1.410(4)	C46-C45	1.404(5)
O8$^{\#1}$-Zn1-N4	113.04(1)	C36-N5-Zn1	127.9(2)	O4-C9-C4	124.7(3)	O2-C1-C2	120.2(3)
O8$^{\#1}$-Zn1-N5	121.23(1)	C48-N7-C41	125.8(3)	O3-C9-C4	111.1(3)	C15-C14-C13	120.3(3)
O12-Zn1-O8$^{\#1}$	118.16(9)	C48-N7-C50	107.7(3)	C25-C24-C23	119.0(3)	C26-C25-N3	119.1(3)
O12-Zn1-N4	98.65(1)	C50-N7-C41	126.5(3)	C27-C22-N2	118.9(3)	C24-C25-N3	118.1(3)
O12-Zn1-N5	105.23(1)	C35-N6-C38	123.9(3)	C23-C22-N2	118.5(3)	C24-C25-C26	122.7(3)
N5-Zn1-N4	96.22(1)	C35N637	106.9(3)	C23-C22-C27	122.6(3)	C22-C23-C24	120.3(3)
O5$^{\#2}$-Zn2-N1	120.13(1)	C4-C5-C6	119.9(3)	C20-C21-N2	105.8(3)	C41-C42-C43	118.6(3)
O5$^{\#2}$-Zn2-N8$^{\#3}$	105.79(1)	C16-C11-C10	121.4(3)	C31-C30-C29	120.3(3)	C41-C42-C47	123.6(3)
O1-Zn2-O5$^{\#2}$	112.91(9)	C12-C11-C10	119.1(3)	O9-C18-C13	113.0(3)	C47-C42-C43	117.8(3)
O1-Zn2-N1	98.56(1)	C12-C11-C16	119.4(3)	O10-C18-O9	123.0(3)	C39-C38-N6	117.8(3)
O1-Zn2-N8$^{\#3}$	116.37(1)	C28-C29-C30	119.9(3)	O10-C18-C13	124.0(3)	C43-C38-N6	119.9(3)
N1-Zn2-N8$^{\#3}$	103.23(1)	C7-C2-C1	120.6(3)	N5-C35-N6	111.4(3)	C39-C38-C43	122.3(3)
C17-O8-Zn1$^{\#2}$	116.88(2)	C3-C2-C7	119.1(3)	C37-N6-C38	129.1(3)	C50-C49-N8	110.2(3)
C8-O5-Zn2$^{\#1}$	110.55(2)	C3-C2-C1	120.3(3)	C2-C7-C6	120.7(3)	C42-C41-N7	119.4(3)
C10-O12-Zn1	120.3(2)	O8-C17-C15	116.7(3)	C7-C6-C8	119.8(3)	C40-C41-N7	118.6(3)
C1-O1-Zn2	115.4(2)	O7-C17-O8	123.3(3)	C7-C6-C5	119.6(3)	C40-C41-C42	122.0(3)
C32-N4-Zn1	122.2(2)	O7-C17-C15	120.0(3)	C5-C6-C8	120.6(3)	C38-C43-C42	117.3(3)

C32-N4-C33	106.3(3)	C4-C3-C2	120.8(3)	O12-C10-C11	115.0(3)	C44-C43-C42	119.2(3)
C33-N4-Zn1	131.5(2)	C16-C15-C17	120.7(3)	O11-C10-O12	125.3(3)	C44-C43-C38	123.6(3)
C19-N1-Zn2	120.7(2)	C14-C15-C16	119.4(3)	O11-C10-C11	119.7(3)	C37-C36-N5	109.2(3)
C19-N1-C20	106.0(3)	C14-C15-C17	119.6(3)	N1-C19-N2	110.4(3)	C30-C31-C26	121.7(3)
C20-N1-Zn2	132.6(2)	C25-C26-C27	117.8(3)	C21-C20-N1	109.9(3)	C49-C50-N7	105.6(3)
C48-N8-Zn2#4	124.8(2)	C31-C26-C25	124.5(3)	N4-C32-N3	111.1(3)	C34-C33-N4	109.0(3)
C48-N8-C49	105.3(3)	C31-C26-C27	117.8(3)	N8-C48-N7	111.2(3)	C33-C34-N3	106.4(3)
C49-N8-Zn2#4	124.1(2)	C14-C13-C18	121.3(3)	C11-C16-C15	120.7(3)	C41-C40-C39	119.9(4)
C19-N2-C22	124.0(3)	C12-C13-C14	119.6(3)	O5-C8-C6	118.1(3)	C38-C39-C40	120.0(3)
C19-N2-C21	107.9(3)	C12-C13-C18	119.1(3)	O6-C8-O5	122.9(3)	C36-C37-N6	106.6(3)
C21-N2-C22	128.1(3)	C11-C12-C13	120.5(3)	O6-C8-C6	119.0(3)	C45-C44-C43	120.6(4)
C32-N3-C25	127.5(3)	C28-C27-C26	118.9(3)	C5-C4-C9	118.9(3)	C47-C46-C45	120.3(4)
C32-N3-C34	107.2(3)	C28-C27-C22	123.5(3)	C3-C4-C5	119.8(3)	C46-C47-C42	121.7(4)
C34-N3-C25	125.3(3)	C22-C27-C26	117.6(3)	C3-C4-C9	121.3(3)	C44-C45-C46	120.4(4)
C35-N5-Zn1	121.5(2)	C29-C28-C27	121.3(3)	O1-C1-C2	114.9(3)		
C35-N5-C36	105.9(3)	O4-C9-O3	124.2(3)	O2-C1-O1	124.9(3)		

Symmetry transformation: #1-1 + x, y, z; #21 + x, y, z; #31 + x, y, -1 + z; #4-1 + x, y, 1 + z.

Table S3. The selected bond lengths [Å] and angles [°] for CP-2.

Zn1-O5#1	1.942(2)	N1-C11	1.379(4)	C7-C2	1.385(4)	C17-C18	1.428(4)
Zn1-O1	1.942(2)	N4-C25	1.383(4)	C7-C6	1.395(4)	C17-C22	1.419(4)
Zn1-N1	2.004(2)	N4-C23	1.320(3)	C4-C5	1.384(4)	C14-C15	1.409(4)
Zn1-N4#2	2.018(2)	N3-C16	1.444(3)	C4-C3	1.397(4)	C18-C19	1.417(4)
O5-C8	1.285(3	N3-C24	1.380(3)	C2-C3	1.392(4)	C24-C25	1.351(4)
O6-C8	1.246(3	N3-C23	1.335(4)	C2-C1	1.510(4)	C12-C11	1.352(4)
O1-C1	1.284(3)	N2-C13	1.446(3)	C16-C17	1.421(4)	C19-C20	1.364(5)
O3-C9	1.325(3)	N2-C12	1.383(3)	C16-C15	1.352(4)	C22-C21	1.357(4)
O4-C9	1.207(3)	N2-C10	1.336(4)	C6-C5	1.391(4)	C20-C21	1.406(4)
O2-C1	1.234(3)	C8-C6	1.499(4)	C13-C14	1.361(4)	C26-C27	1.444(5)
N1-C10	1.327(3)	C9-C4	1.496(4)	C13-C18	1.413(4)	N5-C27	1.131(5)
O5#1-Zn1-N1	125.44(9)	C12-N2-C13	127.3(2)	C15-C16-N3	118.8(3)	C13-C18-C17	117.7(3)
O5#1-Zn1-N4#2	105.53(9)	C10-N2-C13	124.8(2)	C15-C16-C17	123.1(3)	C13-C18-C19	123.6(3)
O1-Zn1-O5#1	109.59(8)	C10-N2-C12	107.7(2)	C7-C6-C8	118.7(2)	C19-C18-C17	118.7(3)
O1-Zn1-N1	110.60(9)	O5-C8-C6	116.4(2)	C5-C6-C8	122.1(2)	C16-C15-C14	119.2(3)
O1-Zn1-N4#2	97.50(9)	O6-C8-O5	122.7(3)	C5-C6-C7	119.1(3)	C25-C24-N3	105.7(3)
N1-Zn1-N4#2	104.13(1)	O6-C8-C6	120.8(2)	C4-C5-C6	120.5(2)	C11-C12-N2	106.0(3)
C8-O5-Zn1#3	109.92(2)	O3-C9-C4	112.6(2)	C2-C3-C4	119.7(3)	C24-C25-N4	109.9(2)
C1-O1-Zn1	119.06(2)	O4-C9-O3	123.6(3)	O1-C1-C2	114.7(3)	N1-C10-N2	110.8(3)
C10-N1-Zn1	122.4(2)	O4-C9-C4	123.7(3)	O2-C1-O1	124.9(2)	N4-C23-N3	111.5(3)
C10-N1-C11	106.0(2)	C2-C7-C6	120.7(3)	O2-C1-C2	120.3(2)	C20-C19-C18	120.6(3)
C11-N1-Zn1	128.81(2)	C5-C4-C9	119.2(2)	C14-C13N2	117.6(3)	C12-C11-N1	109.5(2)
C25-N4-Zn1#4	127.93(2)	C5-C4-C3	120.0(3)	C14-C13-C18	122.8(3)	C21-C22-C17	120.8(3)
C23-N4-Zn1#4	126.4(2)	C3-C4-C9	120.8(3)	C18-C13-N2	119.6(3)	C19-C20-C21	120.7(3)
C23-N4-C25	105.4(2)	C7-C2-C3	119.8(2)	C16-C17-C18	117.5(3)	C22-C21-C20	120.5(3)

C24-N3-C16	129.2(2)	C7-C2-C1	119.7(2)	C22-C17-C16	123.8(3)	N5-C27-C26	178.7(5)
C23-N3-C16	123.1(2)	C3-C2-C1	120.5(3)	C22-C17-C18	118.7(3)		
C23-N3-C24	107.6(2)	C17-C16-N3	118.0(3)	C13-C14-C15	119.4(3)		

Symmetry transformation: #1-1/2 + x, 3/2 - y, -1/2 + z; #23/2 - x, 1/2 + y, 1/2 - z; #31/2 + x, 3/2- y, 1/2 + z; #43/2 - x, -1/2 + y, 1/2 - z.

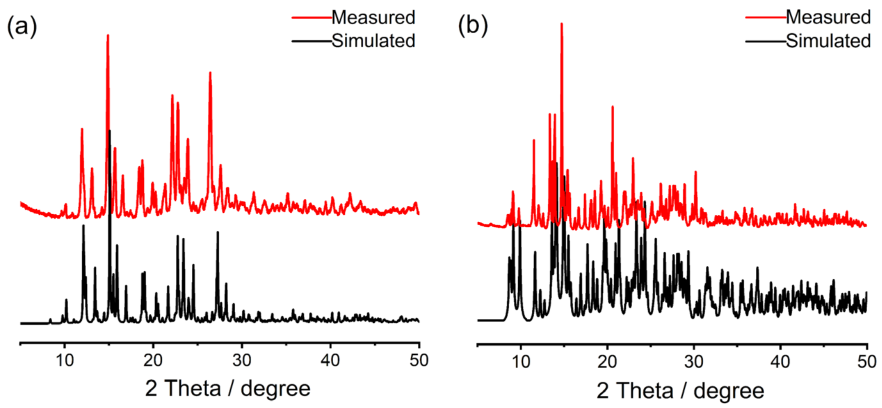

Figure S1. PXRD patterns of simulated and as-synthesized CP-1 and CP-2.

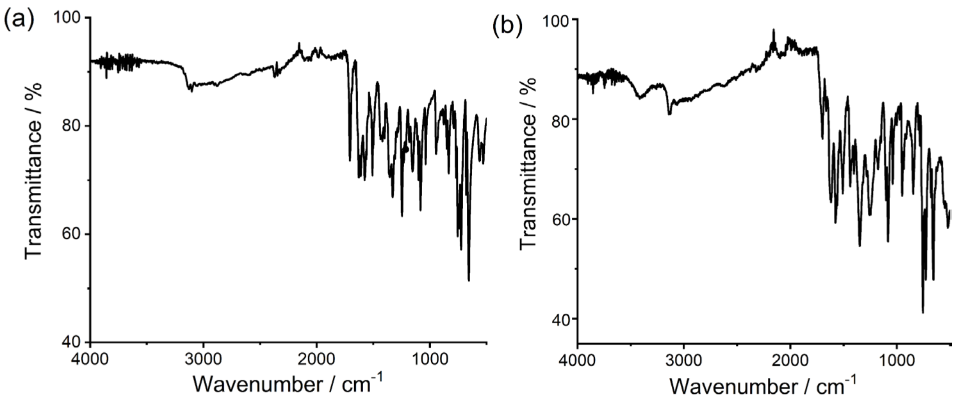

Figure S2. FT–IR spectra of CP-1 and CP-2.

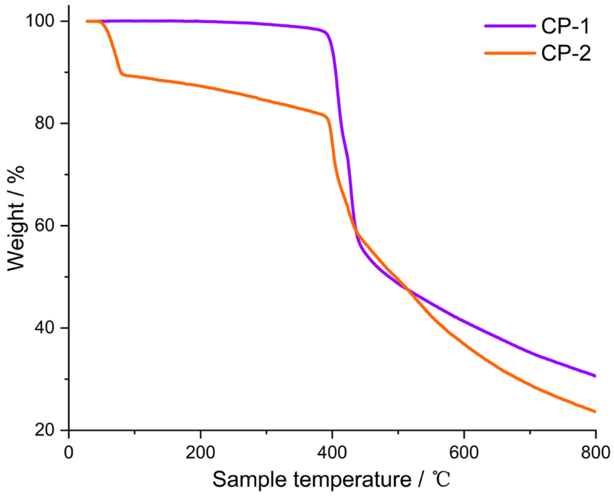

Figure S3. TGA spectra of CP-1 and CP-2.

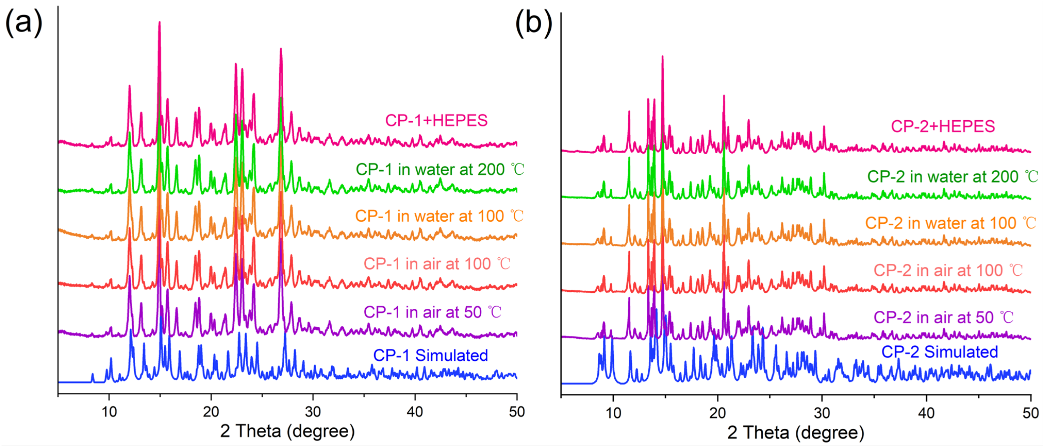

Figure S4. PXRD patterns of CP-1 (a) and CP-2 (b) under different conditions.

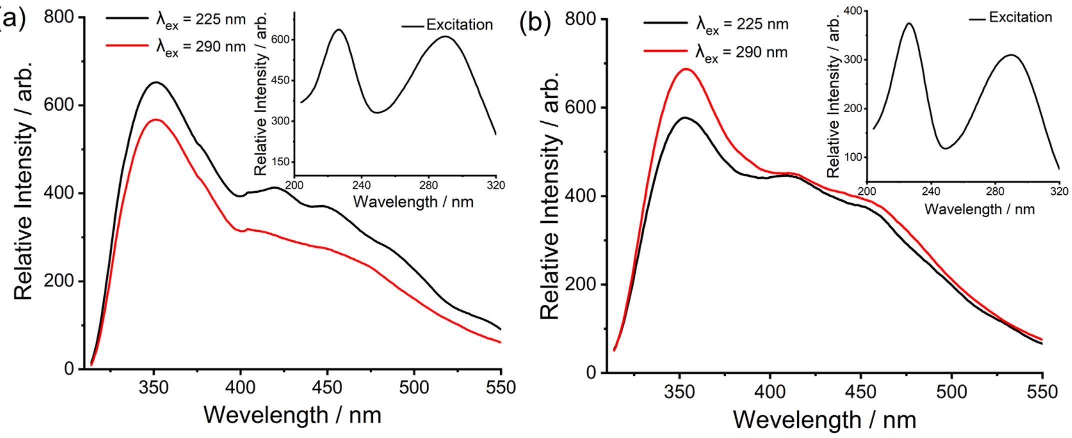

Figure S5. Fluorescence spectra of CP-1 (a) and CP-2 (b) in aqueous suspension at r.t.: excitation (insert) and emission.

pH sensing

To determine a wide range of pH values in aqueous solutions, the ability of the samples was explored. In fluorescence spectra obtained after the gradual addition of sodium hydroxide or concentrated hydrogen chloride to the solution. Interestingly, the fluorescence intensity and maximum emission of CP-1 are closely related to the pH value of the dispersed solution. The fluorescence emission intensity of CP-1 was quenched in the range of pH = 6.2–12.0 (Figure S6) and the maximum emission intensity was linearly dependent on pH in the range of pH = 6.2–12.0. Thus, CP-1 may have the potential ability to quantitatively detect pH values in the range of 6.2 –12.0 in aqueous media.

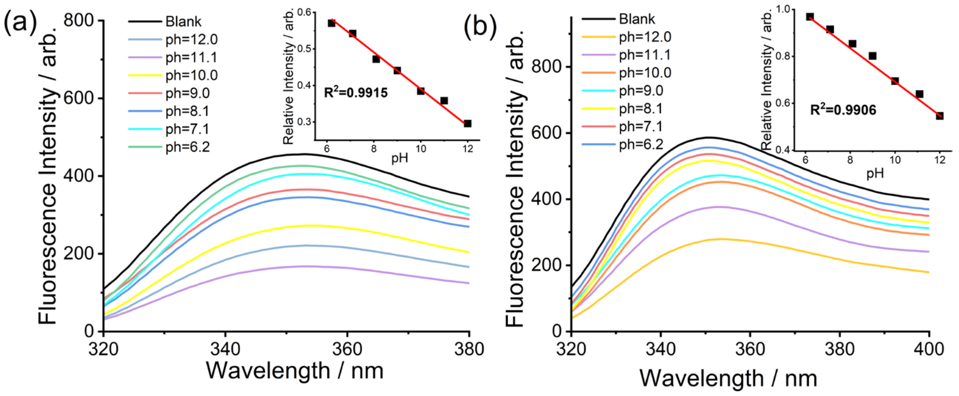

Figure S6. pH-dependent fluorescence and linear variation (insert); (a) excitation of 225 nm (b) excitation of 290 nm.

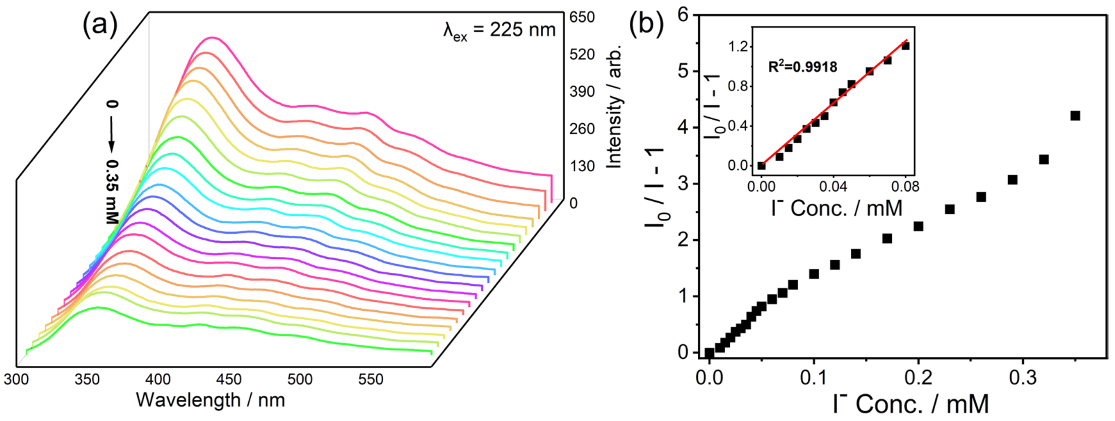

Figure S7. Fluorescence intensities of CP-1 (a) dispersed in different concentrations of I^-; the plot of I_0/I - 1 of CP-1; (b) *vs*. concentration of I^- in aqueous solution (pH = 7.0) at 225 nm (Insert: The plot of I_0/I- 1 of the CPs with the concentration over a I^- concentration range of 0-0.1 mM in aqueous solution).

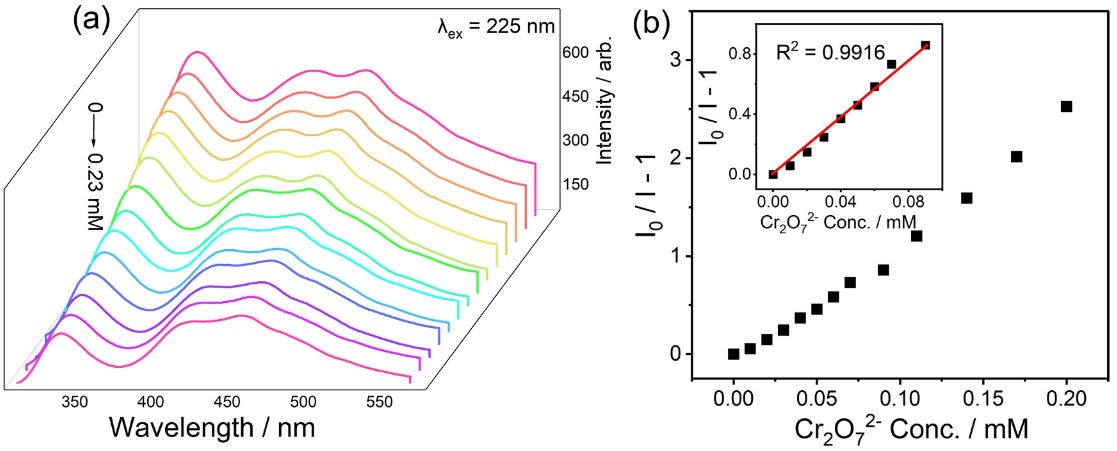

Figure S8. Fluorescence intensities of CP-1 (a) dispersed in different concentrations of $Cr_2O_7^{2-}$; the plot of I_0 / I - 1 of CP-1; (b) *vs*. concentration of $Cr_2O_7^{2-}$ in aqueous solution (pH = 7.0) at 225 nm (Insert: The plot of I_0 / I - 1 of the CPs with the concentration over a $Cr_2O_7^{2-}$ concentration range of 0 - 0.09 mM in aqueous solution).

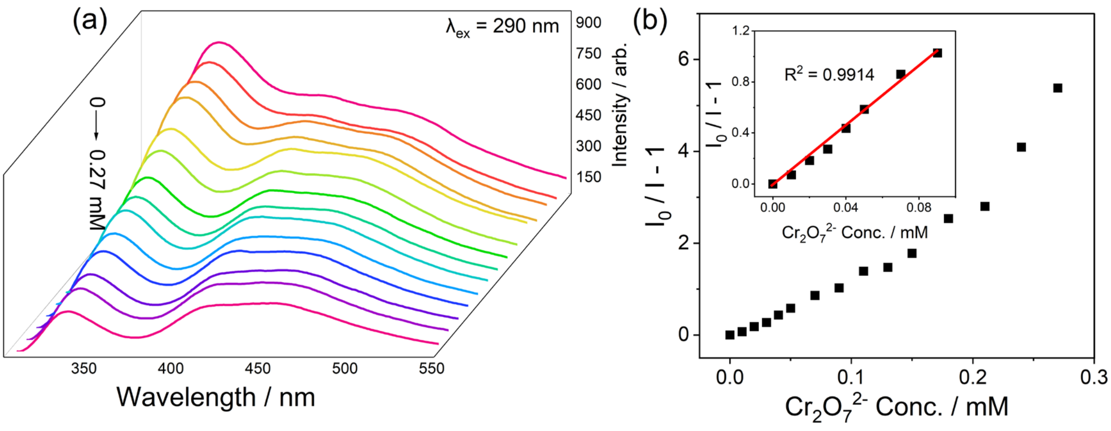

Figure S9. Fluorescence intensities of CP-1 (a) dispersed in different concentrations of $Cr_2O_7^{2-}$; the plot of I_0 / I - 1 of CP-1 (b) *vs*. concentration of $Cr_2O_7^{2-}$ in aqueous solution (pH = 7.0) at 290 nm (Insert: The plot of I_0 / I - 1 of the CPs with the concentration over a $Cr_2O_7^{2-}$ concentration range of 0 – 0.09 mM in aqueous solution).

Sensor	Analyte	Excitation / nm	Emission / nm	Concentration / mg·mL^{-1}	Linear range / μM	LOD / μM	Ksv × 10^4 / M^{-1}
CP-1	I^-	225	350	0.2	0-80	0.33	2.16

Table S4. Ksv and LOD values of CP-1 for ions detection.

Sensor	Analyte	Excitation / nm	Emission / nm	Concentration / mg·mL^{-1}	Linear range / μM	LOD / μM	Ksv × 10^4 / M^{-1}
CP-1	$Cr_2O_7^{2-}$	225	350	0.2	0-90	0.50	1.08
CP-1		290		0.2	0-90	0.44	1.21

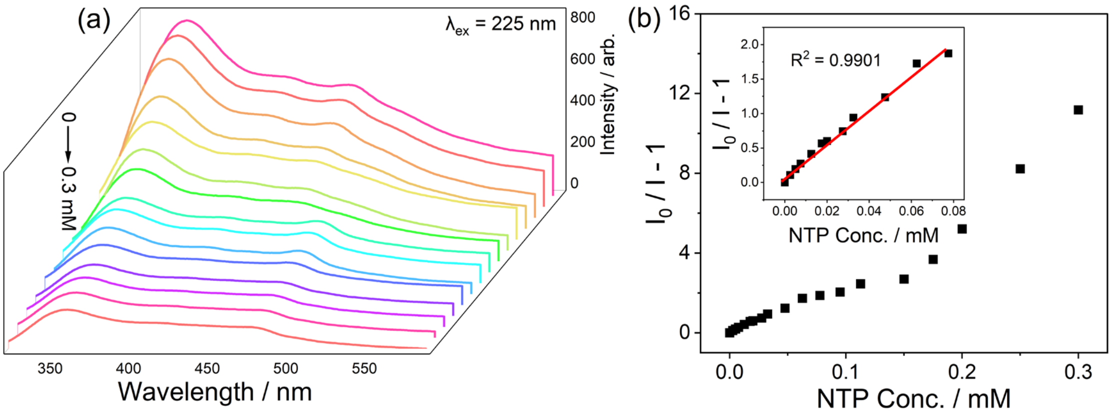

Figure S10. Fluorescence intensities of CP-1 (a) dispersed in different concentrations of NTP; the plot of I_0 / I - 1 of CP-1 (b) *vs*. concentration of NTP in aqueous solution (pH = 7.0) at 225 nm (Insert: The plot of I_0 / I - 1 of the CPs with the concentration over a NTP concentration range of 0 - 0.08 mM in aqueous solution).

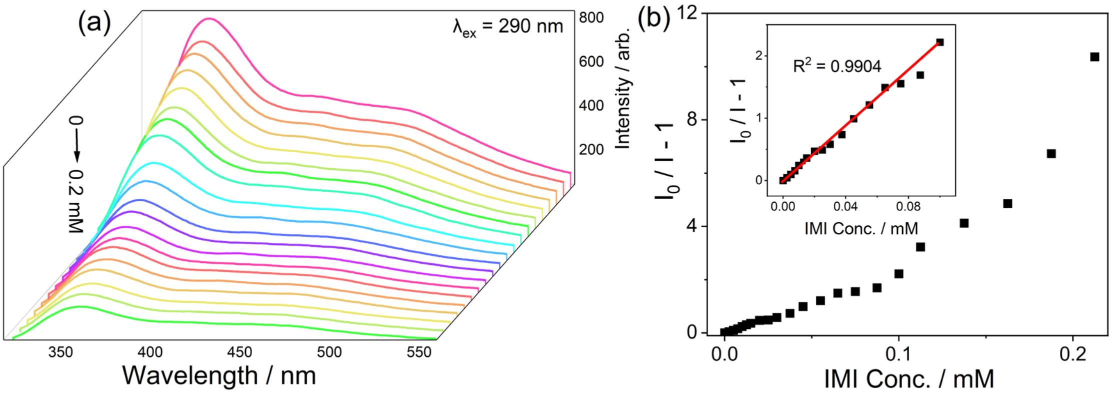

Figure S11. Fluorescence intensities of CP-1 (a) dispersed in different concentrations of IMI; the plot of I_0 / I - 1 of CP-1 (b) *vs*. concentration of IMI in aqueous solution (pH = 7.0) at 290 nm (Insert: The plot of I_0 / I - 1 of the CPs with the concentration over a IMI concentration range of 0 - 0.08 mM in aqueous solution).

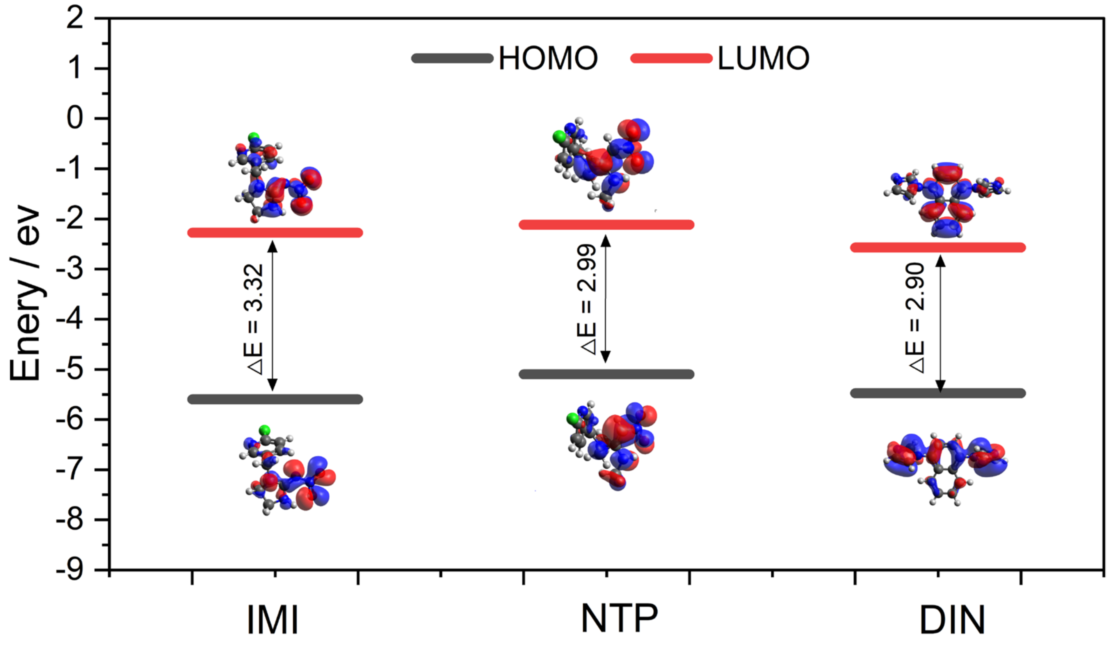

Figure S12. The calculated HOMO and LUMO energy levels of DIN ligand, IMI and NTP.

Table S5. Chemical structures of the selected pesticides.

Name (abbreviation)	Chemical structure
Carbendazim (CAR)	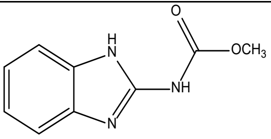
Dipterex (DIP)	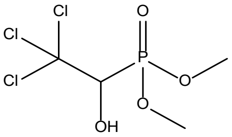
Glyphosate (GLY)	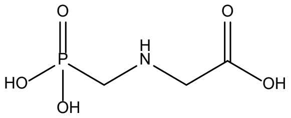
Imidacloprid (IMI)	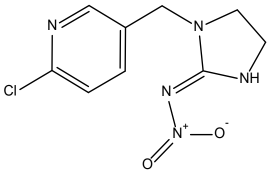
Imazalil (IMZ)	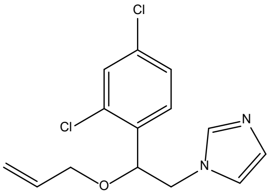
Metamitron (MMT)	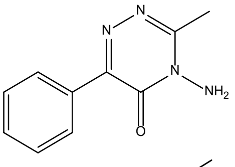
Nitenpyram (NTP)	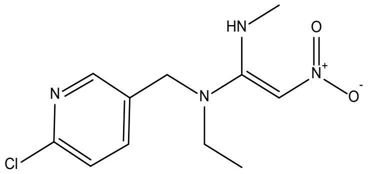
Pentachloro-nitrobenzene (PCNB)	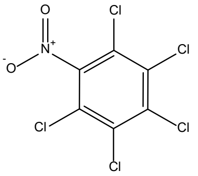
Chlorothalonil (TPN)	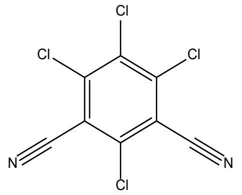
Thiophanate-methyl (TPM)	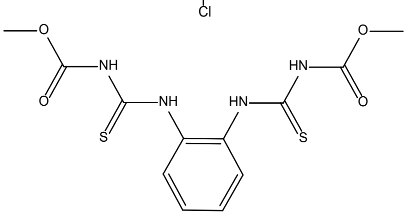
2.4-dichlorophenoxyacetic acid (2,4-D)	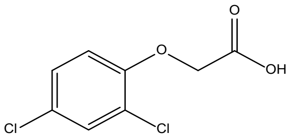

Table S6. Ksv and LOD values for recently reported Zn-MOF-based luminescence probes for sensing of NTP and IMI.

Sensor	λ_{ex} / nm	λ_{em} / nm	Analyte	Concentration / mg·mL^{-1}	Linear range / μM	LOD / μM	Ksv × 10^4 / M^{-1}	Ref.
CP-1	225	350	NTP	0.1	0-80	0.28	3.06	This work
$[Tb(PMBB)_{1.5}(H_2O)_2]$	280	545		1.0	0-100	0.71	1.10	[1]
$[Eu(PMBB)_{1.5}(H_2O)_2]$	280	614		1.0	0-100	0.77	0.99	
$[Cd(NIP)_2(DTP)(H_2O)_2]\cdot H_2O$	250, 290	322		0.1	0-50	0.41	1.87	[2]
$[Cd(NTP)_2(DTP)(Et)(H_2O)_2]\cdot(H_2O)_{0.5}$	250, 290	322		0.1	0-50	0.43	1.79	
$[Cd_3(BTC)_2(DTP)(H_2O)_6]\cdot(H_2O)_{2.5}$	248, 280	322		0.1	0-60	0.27	3.45	[3]
CP-1	290	350	IMI	0.08	0-90	0.25	2.91	This work
$[Zn_3(BTC)_2(DTP)_4(H_2O)_2]\cdot(H_2O)_4$	258	322		0.1	0-50	0.35	2.16	[4]
$[Zn_2(NTD)_2(DTP)]$	258	322		0.1	0-50	0.31	2.49	

1. Zhou, Z. D.; Wang, C. Y.; Zhu, G. S.; Du, B.; Yu, B. Y.; Wang, C. C., Water-stable europium(III) and terbium(III)-metal organic frameworks as fluorescent sensors to detect ions, antibiotics and pesticides in aqueous solutions. *J. Mol. Struct.* **2022**, *1251*, 132009-132019.
2. Zhou, Z.; Li, S.; Wang, W.; Ma, D.; Zhao, H.; Jia, L.; Jia, Y.; Yu, B., Two bis-color excited luminescent sensors of two-dimensional Cd(II)-MOFs bearing mixed ligands for detection of ions and pesticides in aqueous solutions. *J. Mol. Struct.* **2023**, *1273*, 134310-134318.
3. Zhu, G. S.; Cheng, S. L.; Zhou, Z. D.; Du, B.; Shen, Y. Y.; Yu, B. Y., Bisligand-coordinated cadmium organic frameworks as fluorescent sensors to detect ions, antibiotics and pesticides in aqueous solutions. *Polyhedron* **2022**, *217*, 115759-115769.
4. Zhou, Z. D.; Li, S. Q.; Liu, Y.; Du, B.; Shen, Y. Y.; Yu, B. Y.; Wang, C. C., Two bis-ligand-coordinated Zn(II)-MOFs for luminescent sensing of ions, antibiotics and pesticides in aqueous solutions. *RSC Adv.* **2022**, *12*, 7780-7788.
